# Mental well-being during the first months of Covid-19 in adults and children: behavioral evidence and neural precursors

**DOI:** 10.1038/s41598-021-96852-0

**Published:** 2021-09-02

**Authors:** Réka Borbás, Lynn Valérie Fehlbaum, Plamina Dimanova, Alessia Negri, Janani Arudchelvam, Cilly Bernardette Schnider, Nora Maria Raschle

**Affiliations:** 1 Jacobs Center for Productive Youth Development, University of Zurich, Zurich, Switzerland; 2 University Psychiatric Clinics Basel and University of Basel, Basel, Switzerland; 3 Neuroscience Center Zurich, University of Zurich and ETH Zurich, Zurich, Switzerland

**Keywords:** Psychology, Human behaviour

## Abstract

Pandemics such as the Covid-19 pandemic have shown to impact our physical and mental well-being, with particular challenges for children and families. We describe data from 43 adults (31♀, ages = 22–51; 21 mothers) and 26 children (10**♀**, ages = 7–17 years) including pre-pandemic brain function and seven assessment points during the first months of the pandemic. We investigated (1) changes in child and adult well-being, (2) mother–child associations of mental well-being, and (3) associations between pre-pandemic brain activation during mentalizing and later fears or burden. In adults the prevalence of clinically significant anxiety-levels was 34.88% and subthreshold depression 32.56%. Caregiver burden in parents was moderately elevated. Overall, scores of depression, anxiety, and caregiver burden decreased across the 11 weeks after Covid-19-onset. Children’s behavioral and emotional problems during Covid-19 did not significantly differ from pre-pandemic levels and decreased during restrictions. Mothers’ subjective burden of care was associated with children’s emotional and behavioral problems, while depression levels in mothers were related to children’s mood. Furthermore, meeting friends was a significant predictor of children’s mood during early restrictions. Pre-pandemic neural correlates of mentalizing in prefrontal regions preceded later development of fear of illnesses and viruses in all participants, while temporoparietal activation preceded higher subjective burden in mothers.

## Introduction

The global onset of the coronavirus disease 2019 (Covid-19) pandemic has been recognized as a significant threat to our physical and mental well-being. Worldwide efforts have been implemented including protective health measures to slow down or prevent the direct physical effects of the virus. In Switzerland these restrictions included school closure, work-from-home orders, and travel restrictions. Past and accumulating evidence indicates that restrictions (e.g., school closure, lockdown, social distancing) may have a significant effect on individuals’ psychosocial functioning, possibly through increases in emotional distress^1,2^. Evidence indicates that mental health consequences include an increase in neuropsychiatric symptoms of affect and behavior^3,4^. Such increases in negative effects (e.g., stress, anxiety, depression, or somatic complaints) associated with Covid-19 and restrictions are reported globally^1,2,5,6^. The duration of lockdown and restrictions have been linked to increased distress^5^. Negative effects tend to be higher in younger individuals, those with chronic disease or pre-existing health conditions, females and those living alone or in socioeconomic adversity^1,2,7^.

Children’s, parents’, and families’ lives may be particularly impacted by Covid-19-related restrictions^8^. A sudden decrease in social contacts is opposite to the human social nature and our existing routines^9,10^. For children and adolescents, positive peer-relationships, the ability to pursue hobbies and educational opportunities are affected^11^. For parents, an increased burden may result from a disrupted work-life balance. Parental exhaustion, irritability, and mental health symptoms (e.g., depression and anxiety) have been reported to increase during pandemics^12,13^. Moreover, parents’ psychological distress can affect children’s ability to adjust to novel situations and may therefore promote the development of behavioral and emotional problems^14^. High anxiety or depressive symptoms in parents have been associated with an increase in harsh parenting and child abuse potential^15^, indicating urgent consideration for policymakers to provide resources and support for at-risk families.

Notably, reports on increases in emotional distress are complemented by reports of a smaller, but significant, proportion of individuals who describe no changes or increases in well-being during restrictions. Such data indicates that interindividual differences in the effect of restrictions on mental health should be considered^2^. For example, restrictions may bring some families closer together, increase parent–child bonding and joint experiences^7^. An increased understanding of interindividual differences that protect or increase risk for psychopathologies holds the potential to inform personalized support associated with pandemics.

The identification of potential precursors for psychosocial functioning during challenging life events is crucial for the development and implementation of prevention and intervention measures. Socioemotional abilities represent different skill sets of social and emotional functioning^16^ which may serve as potential antecedents of psychosocial functioning during challenging life events^17^. Successful socioemotional skill development in children is positively linked to present and future well-being^18^ and a disruption of these has been linked to externalizing and internalizing problems^19^. Furthermore, socioemotional skill development strongly relies on caregiver-child relationships and dyadic learning^20^.

A fundamental ability for many later-emerging socioemotional abilities is mentalizing, a sociocognitive skill enabling the understanding of emotions, thoughts or motives of others and oneself (enabled by our so-called Theory of Mind and impacted by parenting behaviors^21^). Having a well-developed Theory of Mind has been associated with higher social competences, psychological and physiological functioning^22^. Contrariwise, impaired mentalizing abilities have been linked to stress and depression^23^, potentially serving as a predictor of these^17^. On a neural level, the functional brain network associated with mentalizing typically includes areas such as the bilateral temporoparietal junction, precuneus, medial prefrontal cortex and right superior temporal sulcus^24^, with the temporoparietal junction and prefrontal cortex particularly relevant when thinking about others’ and one’s own mental states^10^. The right temporoparietal junction has been the area most consistently activated during different types of fMRI mentalizing tasks^24^. The right dorsolateral prefrontal cortex is similarly involved during mentalization and perspective taking, but also plays a key role in emotion regulation, which is strongly associated with mental well-being^25,26^. A disrupted ability to mentalize, including associated neural alterations, can be found in clinical disorders, such as borderline personality disorder, conduct disorder or alexithymia^27,28^.

Increasing evidence highlights the urgent need to consider the indirect consequences of the pandemic on physical and psychological well-being. Children’s, parents’, or families’ lives may be particularly affected, and parental well-being is suggested to be intertwined with that of children. Past evidence further indicates that well-being and stress are moderated by sociocognitive skills. In this study, we aimed (1) to investigate the effects of Covid-19 and associated restrictions on child and adult well-being as measured repeatedly during the first months after Covid-19 onset; (2) to assess associations of mental well-being (e.g., anxiety, depression, caregiver burden) in mothers with children’s emotional and behavioral problems or mood; (3) to examine the association between the neural correlates of mentalizing as measured prior to Covid-19 and later development of fear of contamination and illnesses in all participants, or caregiver burden in mothers. In line with prior work^2,29^, we expect reports of negative effects on mental well-being (e.g., general health, anxiety, distress, depression), with possible changes over time. Emotional and behavioral problems in children may vary over time. Furthermore, we suggest that variations in emotional and behavioral problems or mood in children are positively associated with variables of mental well-being of their mothers. In everyday life, increased mentalizing skills are linked to improved socioemotional functioning^22^. However, studies have shown that particularly during challenging life circumstances an elevated tendency to mentalize may also be negatively associated with our well-being (e.g., higher anxiety in those with better mentalization skills^17^). In line with this observation, we suggest that neural correlates of mentalizing are positively associated with later caregiver burden or the development of higher anxiety and fears associated with viruses.

## Methods

### Participants

**Figure 1:**
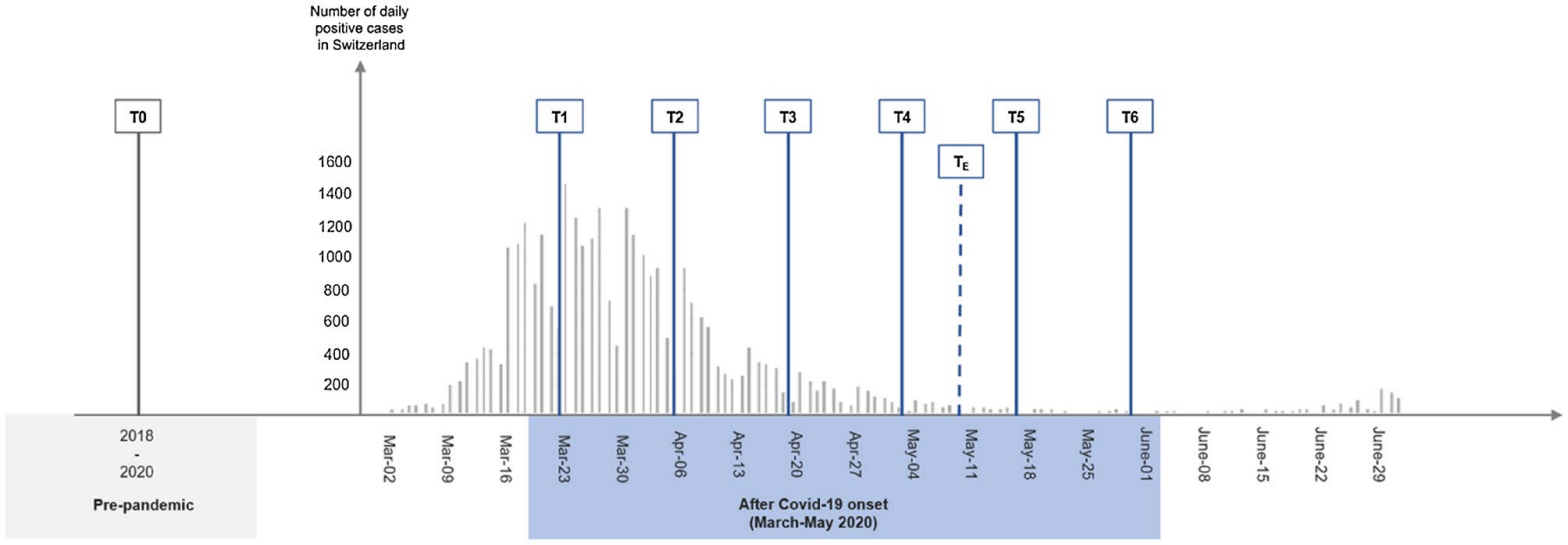
Study design and overview of assessment time points conducted prior to (T0) and during the pandemic and associated restrictive measures in Switzerland (T1–T6).

 Ninety-eight European participants (60 adults and 38 children) of a previous cross-sectional neuroimaging study investigating socioemotional development between 2018 and 2020 were asked to participate in the Covid-19 online follow-up assessments. Pre-pandemic assessments included behavioral tests and functional magnetic resonance imaging (fMRI) during mentalizing; see study description in^30^. We here describe data from the first 3 months after the first implementation of stringent restrictions following Covid-19 onset in Switzerland and include seven assessments time points across this time period (Fig. 1). Sixty-nine participants (43 adults: 31 females; average age = 35.14 years; age range 22–51 years; 26 children: 10 females; average age = 10.69 years; age range 7–17 years) agreed to take part in the follow-up study; retention rate per time point for these 69 individuals were as follows: **T3** (41 adults [95.35%], 24 children [92.31%],); **T4** (39 adults [90.70%], 23 children [88.46%],); **T**_**E**_ (40 adults [93.02%], 24 children [92.31%]); **T5** (29 adults [67.44%], 15 children [57.69%]); **T6** (37 adults [86.05%], 23 children [88.46%]).

All adults and children were previously recruited from the general community and schools for a study on the behavioral and neural correlates of socioemotional skill development. More specifically, participants took part in an evaluation study for a novel cognitive and affective Theory of Mind cartoon task (specifics may be found in^30^). Furthermore, 21 women and 26 children were related (mother–child dyads). Parents of the children reported no known clinical diagnosis for 23 of the children, for three children a clinical diagnosis of ADHD was indicated and for one of these three children the parents further noted a possible developmental delay. In line with guidelines and approval by the local ethics board (Ethikkomission Nordwest- und Zentralschweiz) all participants signed an informed consent form. Additionally, in case of children, verbal assent of the child and written informed consent from a parent and/or legal guardian was collected. All research presented here was performed in accordance with the relevant guidelines and regulations of the Ethikkomission Nordwest- und Zentralschweiz.

### Assessments

Overall, eight testing time points are included, with the first (T0) reporting data obtained during the two years prior to the pandemic. Seven assessments were conducted across 75 days (11 weeks) after Covid-19 onset in Switzerland. The online assessment started following nationwide restrictions implemented in Switzerland on March 16th, 2020, including the ban of events, school closure, closure of all non-essential and hardware stores, garden centers, markets, museums, zoos, nightclubs, closure of hairdresser, restaurants, ban of gatherings (maximum of five people) and home-office orders, etc. Schools were re-opened on May 11th, 2020, resulting in more parents returning to work. Only assessments relevant to the present analyses are described below. Further details, including information for all assessments conducted prior to Covid-19 onset (T0) and during restrictions (T1–T6) may be found in the Supplementary Methods.

Testing prior to Covid-19 (T0) took place between March 2018 and February 2020 and included functional neuroimaging during mentalizing. Online assessments after Covid-19 onset were conducted from March to May 2020. Participants filled out six biweekly online questionnaires (labelled as T1, T2, T3, T4, T5, T6 in Fig. 1). For adults, these targeted *anxiety* (State-Trait Anxiety Inventory or STAI-6; a self-report questionnaire to assess anxiety level as state^31^), *depression* (Center for Epidemiologic Studies Depression Scale or CESD-R, German version^32^; assessing symptoms in the last 1–2 weeks relating dysphoria, anhedonia, appetite, sleep, thinking, guilt, fatigue, movement and suicidal ideation), *general health* (General Health Questionnaire or GHQ-12, German version; a self-report instrument to screen for psychosocial well-being^33^), *distress* (questionnaire adapted from the Kessler Psychological Distress Scale, but answer format was modified allowing participants to indicate their emotional state in relation to their usual emotional state^34^) and *subjective burden of caregiving* for mothers (the Burden Scale for Family Caregivers or BSFC-s^35^; a self-report questionnaire assessing subjective burden of family caregivers, which was adapted to capture increased burden in parental responsibilities during restrictions). In children *emotional and behavioral problems* were assessed using the Strengths and Difficulties Questionnaire (SDQ^36^) and subjective mood ratings (children had to choose between 5 different smileys in order to indicate their mood in the last days. Ratings included 1: very happy, 2: happy, 3: unsure, 4: unhappy, 5: very sad). Children were further asked whether they had met any friends in the previous week. *News consumption* (adults only) and *time spent outside* (all participants) were assessed by asking participants to indicate the amount of time spent on these activities on a 5-point Likert scale. Adults reported their daily news consumption across all forms of media through the following scale: 1: no time, 2: approximately 15 min, 3: approximately 30 min, 4: approximately 1 h, 5: more than an hour of time spent consuming news). Adults and children indicated the average duration of spending time outside per day in the past week (1: no time, 2: half an hour, 3: 1 h, 4: 1–2 h, 5: more than 2 h of time spent outside).

One extra questionnaire (T_E_, between T4 and T5) was added before a first ease in restrictions was introduced by the government. This extra testing consisted of the Child Behavior Checklist (CBCL^37^) evaluating *child behavior* and the *Fear of Illness and Virus Evaluation* (developed by Professor Jill Ehrenreich-May, https://adaa.org/node/5168). CBCL was also acquired at T0 allowing a pre-/post-comparison. Of the six biweekly assessments, the last two (T5, T6) were conducted after schools reopened.

### Behavioral data analyses

#### Mental well-being during Covid-19-related restrictions

First, adults’ scores in anxiety depression, and caregiver burden were screened. STAI-6 total scores above 40 were considered as an indicator of clinically significant levels of anxiety, according to^38^. Depression scores were screened to detect subthreshold depression symptoms according to the CESD total score (CESD_total_ ≥ 16) or meeting criteria for a major depressive episode (description of the algorithm for calculation may be found at: https://cesd-r.com/cesdr/). Next, we calculated the 11-week prevalence of clinically significant anxiety, subthreshold depression and major depression (i.e., the proportion of participants surpassing relevant cut-off scores and fulfilling criteria at least once during the assessment period). Finally, parental burden was classified as “low”, “moderate” or “high” according to the classification suggested by Pendergrass and colleagues^39^ (BSFC-s scores of 0–4 are considered as low; 5–14 as moderate; 15–30 as high).

We investigated the effect of Covid-19 and related restrictions on mental well-being using linear mixed-effect models in R (https://www.r-project.org/). As a first step, missing data points were evaluated to assess whether these were missing at random (MAR). In case of no violation of MAR assumption missing data was replaced by Multivariate Imputation by Chained Equations MICE package in R^40^ employing the predictive mean matching method. Overall, 14.41% of the testing time points reported in the present analyses were imputed (12.79% in adults, 16.03% in children).

Linear mixed-effects models were employed to analyze the relationship between length since Covid-19 onset and continuous outcome measures (depression, anxiety, general health, distress, caregiver burden, and emotional and behavioral problems in children) using lme4^41^. Duration (in weeks) was entered as a fixed effect. Subjects were entered as a random effect and the model allowed for random intercepts and random slopes accounting for non-independence of datapoints (same person answering multiple times). Furthermore, a different response of the subjects was expected (each person might react differently to duration of restrictions). *P* values were obtained by the Satterthwaite approximation as recommended by Luke et al.^42^ for small group sizes using the lmerTest package^43^. This pipeline was adjusted for the analysis of depression, caregiver burden and emotional and behavioral problems in children for the following reasons: Depression scores (CESD-R) and children’s emotional problems, conduct problems, hyperactivity, peer problems and total scores (SDQ) were log-transformed after a visual inspection of the data revealing a right skew. For caregiving burden (BSFC-s), and children’s peer problems and total score of emotional and behavioral problems (SDQ), the full model (including random intercepts and slopes for each subject) indicated an overfit. Consequently, a simplified model excluding random slopes by subject was implemented.

For the analysis of categorical, non-parametric data (i.e., clinically relevant threshold for depression reached [yes/no], time spent outside, news consumption and mood in children), Friedman tests were used. Significant main effects were followed up using post-hoc pairwise comparisons and adjusted using Holm-Bonferroni correction. Finally, one-way analysis of variance was employed to test whether emotional and behavioral problems (SDQ and CBCL) in children differed prior to and during Covid-19-related restrictions. For the score during Covid-19 all time points of SDQ were averaged to build one score (average of five online assessments). CBCL was only assessed once at T_E_.

#### Mother–child associations

To test whether mental well-being in mothers (anxiety, depression, and caregiver burden) explained variability in children’s emotional or behavioral problems a multiple regression analysis was implemented corrected for children’s age and sex. Since emotional and behavioral problems in children were assessed through parental reports, parental bias may impact findings. Therefore, we repeated the multiple regression analysis by using mood scores provided by the children as a dependent variable.

#### Post-hoc follow-up assessment

Mental well-being and the development of negative symptoms during stressful life events have been suggested to be influenced by further variables of interest, including sex and parenting^44^, news exposure^2^ or time spent outside^45^. For adult participants, multiple regression analysis controlling for age was conducted to assess whether variation in mental well-being (i.e., anxiety, depression, or distress) were explained by sex, news consumption, time spent outside or parenthood. For children, we assessed whether children’s well-being (self-report for mood) during restrictions was explained by time spent outside or meeting friends (yes/no) using multiple regression analyses, controlling for age and sex of the children.

#### Children’s subjective reports

Children were asked two open-ended questions: At T1–T4, these were “What do you like about spending more time at home now?” and “What do you like less about spending more time at home now?”. At T5 (after the first week of school opening) and T6 (3 weeks after school reopened) these were changed to “What do you like about going back to school?” and “What do you like less or think, is a bit annoying, about going back to school?” Subcategories based on topics mentioned were built and coded by two independent reviewers (Supplementary Methods).

### fMRI data analyses

fMRI data was analyzed using SPM12 running on MATLAB R2020b (www.fil.ion.ucl.ac.uk/spm). Neural correlates of mentalizing were tested using the CAToon task^30^ (see^30^ and Supplementary Methods). fMRI was acquired for all participants between 2018 and 2020. In short, fMRI during mentalization was acquired using a cartoon-based Theory of Mind task [experimental condition: affective (AT) and cognitive (CT) Theory of Mind; control condition: physical causality (PC)]. The neural correlates of mentalizing were based on a regressor of interest including both cognitive and affective Theory of Mind as compared to physical causality ((AT|CT) > PC). Whole-brain T2-weighted echo-planar images were collected using a 20-channel head coil on a Siemens 3T Prisma MR scanner (specifics in Supplementary Methods). Group analyses included age and sex as covariates and all findings were corrected for multiple comparisons using whole brain family-wise error correction (FWE).

For the present purpose mean parameter estimates were extracted for areas of interest consistently recruited during mentalizing^24^, including right temporoparietal junction (TPJ) and dorsolateral prefrontal cortex (dlPFC), using the MarsBar toolbox^46^. More specifically, right TPJ was selected as a region of interest since it is most consistently recruited during mentalizing tasks and perspective taking in both children and adults^47^. A 7 mm sphere was extracted for the right TPJ, because the group activation cluster extended beyond the area of interest (spanning over 5860 voxels reaching from temporal pole to occipital areas). The right dlPFC was selected as a region of interest, because of its involvement during mentalization and perspective taking, but also because of its key role in emotion regulation, which is in turn strongly associated with mental well-being^25^, including the development of stress-related burden, depression and anxiety^26,48^. To test whether these regions were significant predictors of fears about contamination and illness, or caregiver burden, we employed multiple regression analyses controlling for age and sex when applicable. For the multiple regression analysis including caregiver burden we calculated one score averaging all BSFC_total_ scores. In-scanner data collection was only evaluated to assure task compliance (i.e., no more than 10% missing in all trials; Supplementary Table S1).

## Results

### Behavioral findings

#### Descriptive statistics

**Figure 2:**
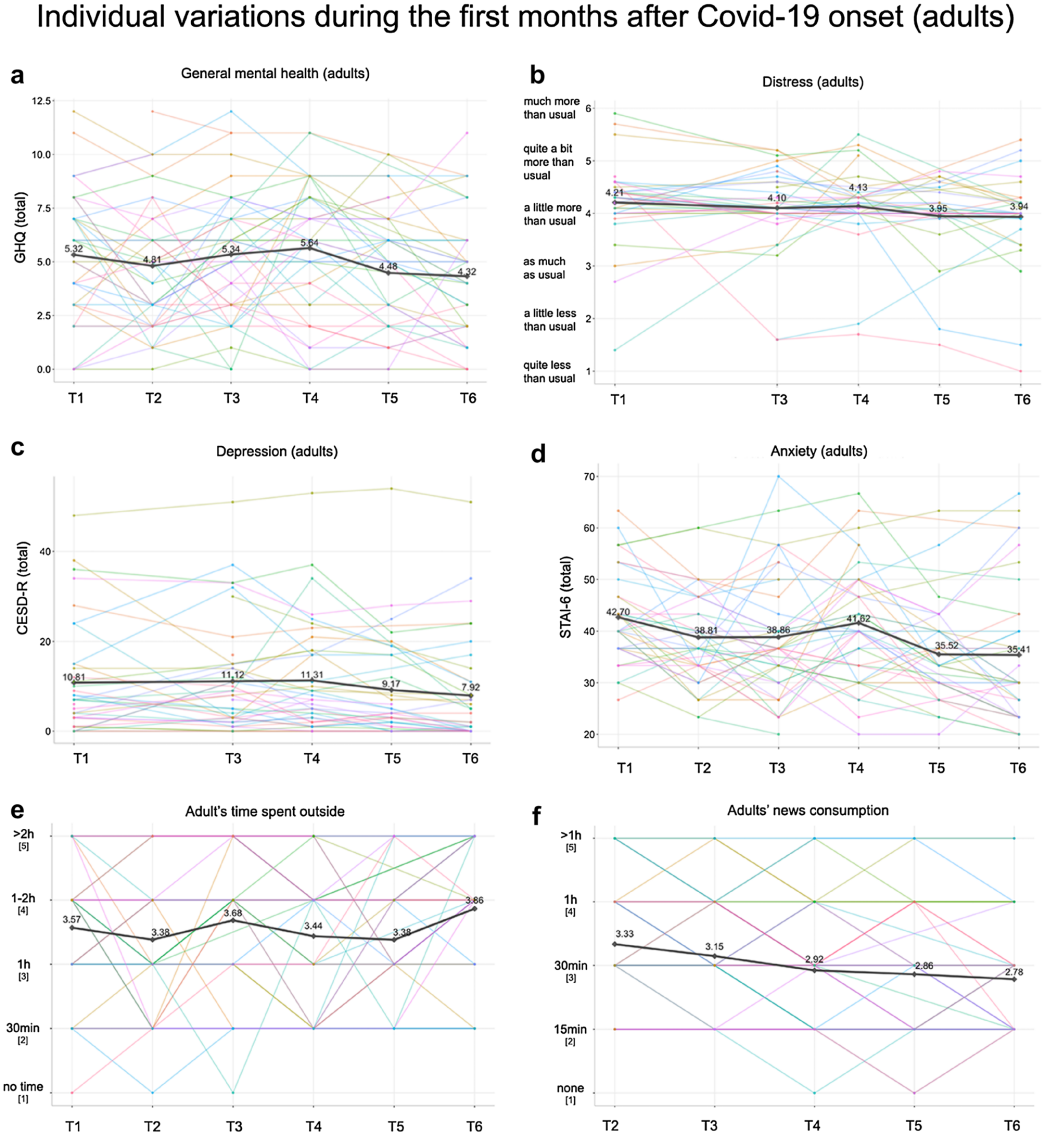
Variations of group mean (bold) and individual (colorful) scores in mental well-being across the first months after Covid-19 onset in adults. (**a**) Variation in scores of general mental health. (**b**) Variation in distress scores. (**c**) Variation in depression scores. (**d**) Variation in anxiety scores. (**e**) Variation in time spent outside. (**f**) Variation in news consumption.

A summary of the behavioral data collected prior to and during the early weeks following Covid-19 onset is included in Table 1 (in children scores prior to and scores averaged over the 11-weeks online assessment are reported. For adults only averaged scores are reported; Fig. 2).

**Table 1:** Group characteristics of adults and children prior to and during the first months after Covid-19 onset.

	Adults (n = 43, 31 females)		Children (n = 26, 10 females)					
	First pandemic months	M ± SD		Pre-pandemic	M ± SD		First pandemic months	M ± SD
Age	In years	35.14 ± 9.20	Age	In years	9.58 ± 2.39	Age	In years	10.69 ± 2.52
Time s. 1st test	In months	18.76 ± 7.03	IQ	Verbal	13.88 ± 8.94	Time s. 1st test	In months	13.64 ± 7.01
ISCED		4.84 ± 1.75		Non-verbal	12.88 ± 4.48	SDQ^a^	Emotional problems	1.21 ± 1.62
BSFC^ab^	Subjective burden of care	8.32 ± 4.42	SDQ	Emotional problems	1.73 ± 2.24		Conduct problems	1.64 ± 1.49
STAI-6^a^	Anxiety	38.85 ± 8.57		Conduct problems	1.69 ± 1.72		Hyperactivity	2.88 ± 1.93
Distress^ac^	Distress	4.09 ± 0.56		Hyperactivity	2.81 ± 1.86		Peer problems	1.64 ± 1.44
GHQ^a^	Mental health	5.15 ± 2.57		Peer problems	0.92 ± 1.41		Prosocial	6.56 ± 1.53
CESD-R^b^	Depression	9.96 ± 10.60		Prosocial	7.35 ± 1.67		Total	7.38 ± 4.87
				Total	7.15 ± 4.97	CBCL	Withdrawn	54.58 ± 5.38
News	[1] no time	1.89%	CBCL ^d^	Withdrawn	54.27 ± 5.50		Somatic problems	56.54 ± 7.46
Consumption^a^	[2] 15 min	36.04%		Somatic problems	55.46 ± 5.57		Anxious/depressed	55 ± 8.32
(daily)	[3] 30 min	30.76%		Anxious/depressed	56.73 ± 8.49		Social problems	53.13 ± 4.78
	[4] 1 h	21.82%		Social problems	53.65 ± 4.63		Schizoid-compulsive	54.13 ± 6.49
	[5] > 1 h	9.49%		Schizoid-compulsive	54.35 ± 6.36		Attention problems	55 ± 5.82
				Attention problems	55.19 ± 5.84		Delinquent behaviour	52.38 ± 4.43
Time outside^a^	[1] No time	1.25%		Delinquent behaviour	52.69 ± 3.90		Aggressive behaviour	53.29 ± 5.29
(daily)	[2] 30 min	21.78%		Aggressive behaviour	55.38 ± 6.83		Total	51 ± 9.36
	[3] 1 h	19.77%		Total	53.81 ± 8.45	FIVE	Fears about contamination and illness	12.38 ± 2.78
	[4] 1–2 h	34.93%					Fears about social distancing	15.17 ± 4.27
	[5] > 2 h	22.28%					Behaviors related to illness and viruses	29.63 ± 5.32
FIVE	Fears about contamination and illness	13.53 ± 2.94					Impact of illness and virus fears	2.83 ± 1.01
	Fears about social distancing	15.10 ± 3.63					Total	30.38 ± 6.76
	Behaviors related to illness and viruses	30.55 ± 4.85				Time outside^a^	[1] No time	0.72%
	Impact of illness and virus fears	2.98 ± 1.05				(daily)	[2] 30 min	12.79%
	Total	31.6 ± 6.11					[3] 1 h	18.49%
							[4] 1–2 h	32.90%
							[5] > 2 h	35.10%
						Mood^a^	[1] Very happy	31.34%
							[2] Happy	46.07%
							[3] Unsure	15.44%
							[4] Unhappy	5.70%
							[5] Very sad	1.45%

*Time s. 1st test* time since first testing, *ISCED* international standard classification of education, *BSFC* burden scale for family caregivers, *STAI-6* state-trait anxiety inventory, *GHQ* general health questionnaire, *CESD-R* center for epidemiologic studies depression scale, *FIVE* fear of illness and virus evaluation, *SDQ* strengths and difficulties questionnaire, *CBCL* child behavior checklist.

^a^Average score.

^b^In mothers only.

^c^Distress: 1—much less than usual, 2—quite less than usual, 3—a little less than usual, 4—as much as usual, 5—a little more than usual, 6—quite a bit more than usual, 7—much more than usual.

^d^N = 25 (out of a total N pre-/during confinement of 26).

#### Well-being during Covid-19 in adults

**Figure 3:**
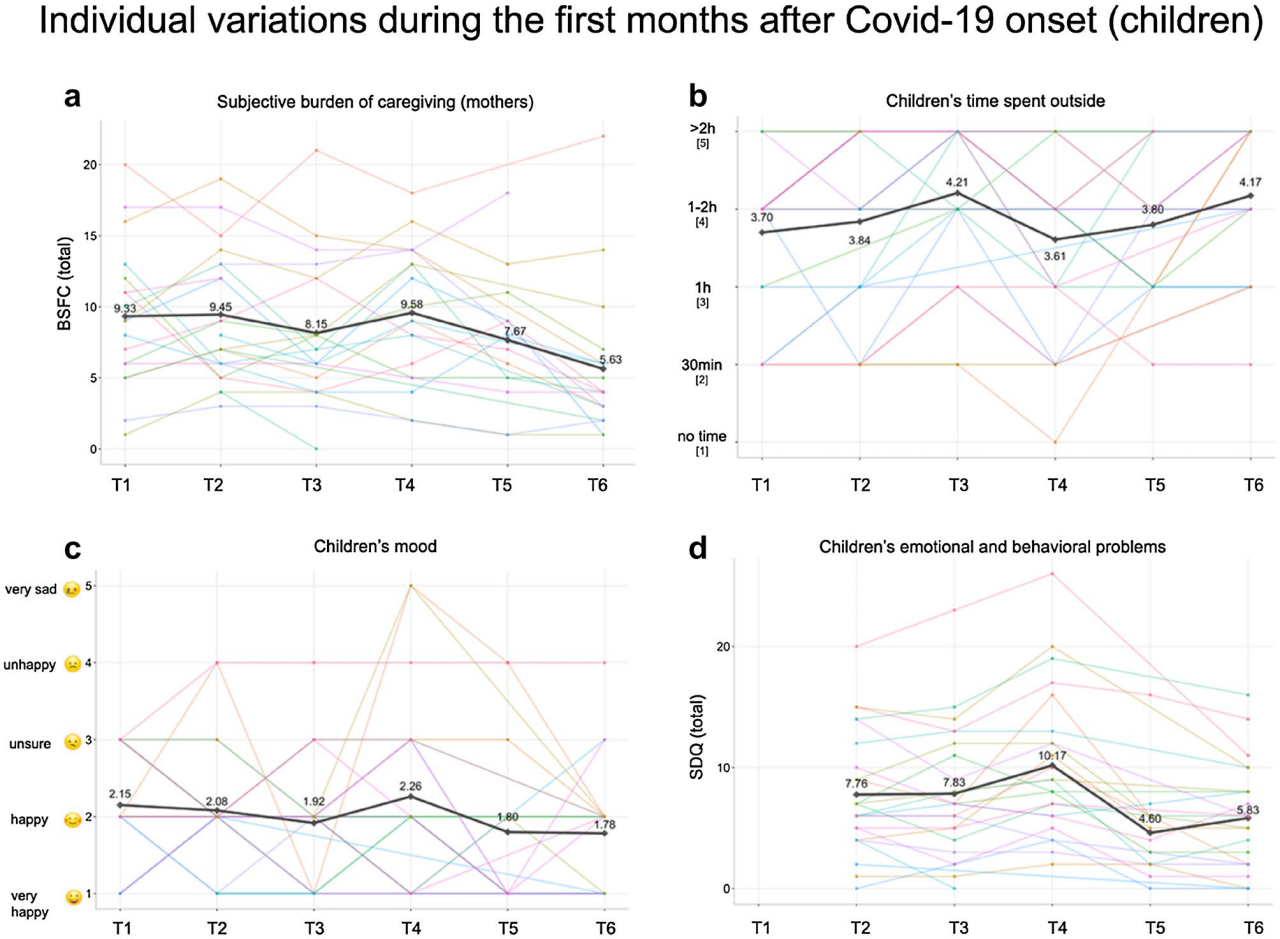
Variations of group mean (bold) and individual (colorful) scores in mental well-being across the first months of Covid-19 onset in children and mothers. (**a**) Variation in mothers' subjective burden. (**b**) Variation in children’s time spent outside. (**c**) Variation in children’s mood. (**d**) Variation in children’s emotional and behavioral problems.

32.56% of all adults reported increased depression scores indicating the presence of subthreshold depressive symptoms (CESD_total_ ≥ 16) with 4.65% meeting the criteria for a major depressive episode at least once. The prevalence of clinically significant anxiety was 34.88%. Group average scores reached clinically significant levels of anxiety at T1 (mean = 42.70, SD = 8.952) and T4 (mean = 41.62, SD = 8.798). Group average scores of subjective burden were in the moderate range (BSFC-s scores of 5–14^39^) throughout the whole assessment period (Fig. 3).

When estimating the effect of restrictions on mental health longitudinally, linear mixed-effect models revealed a small but significant decrease in depression (β = − 0.04), anxiety (β = − 0.61), and burden of caregiving (β = − 0.26) scores with each week passing by. There was a non-significant decrease in general health (β = − 0.06) and distress (β = − 0.02) scores. A detailed summary of all models is included in Table 2.

**Table 2:** Linear mixed models in adults estimating the effect of time after Covid-19 onset on mental health indices.

Predictors	CESD (log)			STAI			BSFC			GHQ			Distress		
	Estimates (SE)	CI (95%)	*p*	Estimates (SE)	CI (95%)	*p*	Estimates (SE)	CI (95%)	*p*	Estimates (SE)	CI (95%)	*p*	Estimates (SE)	CI (95%)	*p*
Intercept	2.05 (0.17)	1.72–2.37		42.58 (1.41)	39.82–45.33		10.07 (0.97)	8.16–11.98		5.49 (0.43)	4.65–6.34		4.22 (0.12)	3.99–4.46	
Duration (weeks)	− 0.04 (0.02)	− 0.07 to − 0.01	**0.012**	− 0.61 (0.16)	− 0.93 to − 0.29	**0.001**	− 0.26 (0.08)	− 0.42 to − 0.09	**0.003**	− 0.06 (0.04)	− 0.13 to 0.02	0.162	− 0.02 (0.02)	− 0.05 to 0.01	0.218
ICC	0.78			0.59			0.56			0.59			0.67		
N	215/43			258/43			132/22			258/43			215/43		

*CESD* center of epidemiologic studies depression scales, *STAI* state and trait anxiety inventory (state anxiety sum scores), *BSFC-s* burden scale for family caregivers (sum score), *GHQ* General Health Questionnaire (sum score), *Distress* modified Kessler psychological distress scale (mean), *SE* standard error, *CI* confidence interval, *Duration (weeks)* fixed effect, weeks passed since restrictions have been introduced, *ICC* intraclass correlation coefficient, *N* number of observations)/(number of participants), *p* values have been estimated using Satterthwaite approximation, significant effects in bold.

For the categorical variables Friedman test of differences revealed significant variations in *time spent outside* (χ^2^ = 18.422, *p* = 0.002) and *news consumption* (χ^2^ = 25.177, *p* < 0.001). Follow-up Bonferroni-corrected pairwise comparisons showed no significant differences for time spent outside between timepoints. For news consumption, follow-up pairwise comparisons showed significant differences between timepoints T2 and T6 (Fig. 2).

#### Well-being during Covid-19 in children

Linear mixed-effects models indicated a significant decrease in children’s scores of conduct problems (β = − 0.04), hyperactivity (β = − 0.03), peer problems (β = − 0.03) and overall emotional and behavioral problems (β = − 0.04; total score of SDQ), whereas there was a non-significant decrease in emotional problems (β = − 0.003) and increase in prosocial behavior (β = 0.08). A detailed summary of all models is included in Table 3. Friedman test revealed a significant variation in *time spent outside* (χ^2^ = 21.002, *p* < 0.001), with significant differences between timepoints T1 and T3. A significant variation over time was also revealed in *mood* ratings (χ^2^ = 13.425, *p* = 0.020), however, post-hoc pairwise comparisons remained non-significant. One-way analysis of variance indicated no significant difference in behavioral and emotional problems in children when comparing pre-Covid-19 scores with average scores obtained during Covid-19 (Supplementary Table S2).

**Table 3:** Linear mixed models estimating the effect of time after Covid-19 onset on children’s behavioral and emotion problems.

Predictors	Conduct problems (log)			Emotional problems (log)			Hyperactivity (log)			Peer problems (log)			Prosocial behavior			Total (log)		
	Estimates (SE)	CI (95%)	*p*	Estimates (SE)	CI (95%)	*p*	Estimates (SE)	CI (95%)	*p*	Estimates (SE)	CI (95%)	*p*	Estimates (SE)	CI (95%)	*p*	Estimates (SE)	CI (95%)	*p*
Intercept	1.02 (0.15)	0.73–1.32		0.55 (0.17)	0.23–0.88		1.38 (0.14)	1.10–1.67		1.04 (0.14)	0.77–1.31		5.96 (0.46)	5.06–6.86		2.20 (0.15)	1.91–2.49	
Duration (weeks)	− 0.04 (0.01)	− 0.07 to − 0.01	**0.012**	− 0.003 (0.02)	− 0.03 to 0.03	0.834	− 0.03 (0.01)	− 0.05 to − 0.00	**0.047**	− 0.03 (0.01)	− 0.06 to − 0.01	**0.016**	0.08 (0.05)	− 0.00 to 0.17	0.075	− 0.04 (0.01)	− 0.07 to − 0.02	**0.001**
ICC	0.77			0.49			0.72			0.56			0.58			0.66		
N	130/26			130/26			130/26			130/26			130/26			130/26		

*SE* standard error, *CI* confidence interval, *Duration (weeks)* fixed effect, weeks passed since restrictions have been introduced, *ICC* intraclass correlation coefficient, *N* (number of observations)/(number of participants), *p* values have been estimated using Satterthwaite approximation, significant effects in bold.

#### Mother–child associations

The multiple regression analyses including age and sex of the children revealed that the full model for mothers’ subjective burden of caregiving explained 52.7% (ß = 0.763, *t*(22) = 4.762, *p* < 0.001) of the variance in children’s emotional and behavioral problems (complete model: *F*(3,22) = 8.173, *p* < 0.001; R^2^ = 0.527 [adjusted R^2^ = 0.463]). Anxiety and depression in mothers did not enter the model. Children’s self-reported mood was best predicted by mothers’ depression scores (ß = 0.660, *t*(22) = 4.136, *p* < 0.001). Depression scores explained 45.2% of variance in children’s mood (complete model including depression, age and sex: *F*(3,22) = 6.037, *p* = 0.004; R^2^ = 0.452 [adjusted R^2^ = 0.377]). Mothers’ experienced burden of caregiving and anxiety did not enter the final model.

#### Post-hoc follow-up assessments

Post-hoc multiple regression analyses revealed no impact of sex, news consumption, time spent outside or parenthood on variations in scores of anxiety, depression or distress in adults, as neither entered into the prediction model. For children, meeting friends (yes/no) explained 35.5% of the variation and entered into the model as a significant predictor of mood (ß = − 0.601, *t*(22) = − 3.551, *p* = 0.002). Mood was negatively coded (lowest score representing the best mood and highest scores representing lowest mood/sadness), indicating that meeting friends was positively linked to a better mood. The model including meeting friends controlling for age and sex was established as a significant predictor of mood with an R^2^ = 0.380 (adjusted R^2^ = 0.294; *F*(3, 22) = 4.499, *p* = 0.013).

#### Children’s qualitative reports

**Figure 4:**
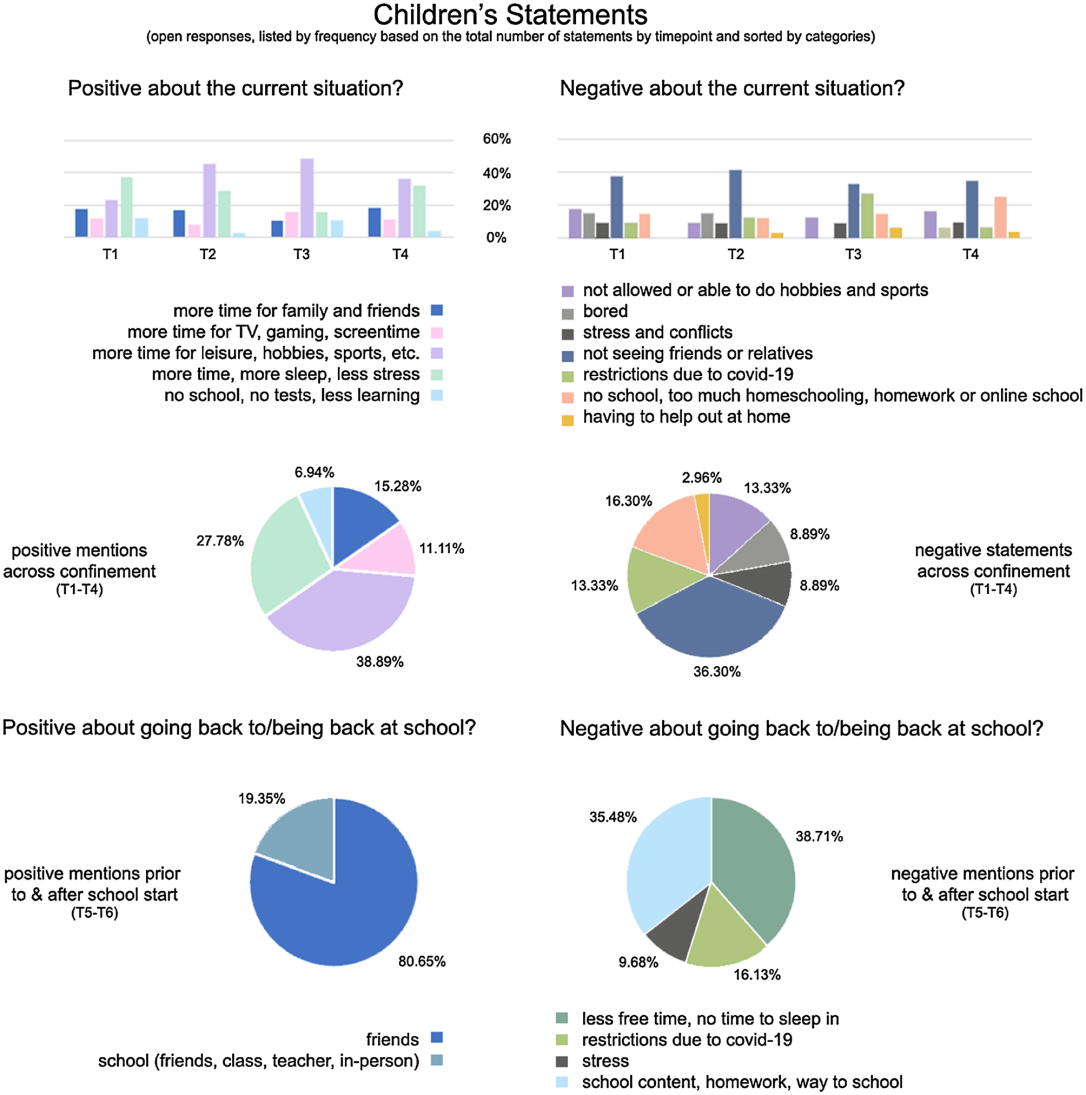
Qualitative measures of positive and negative associations with school closure or opening in children.

An overview about children’s subjective statements is given in Fig. 4.

### Neuroimaging findings

**Figure 5:**
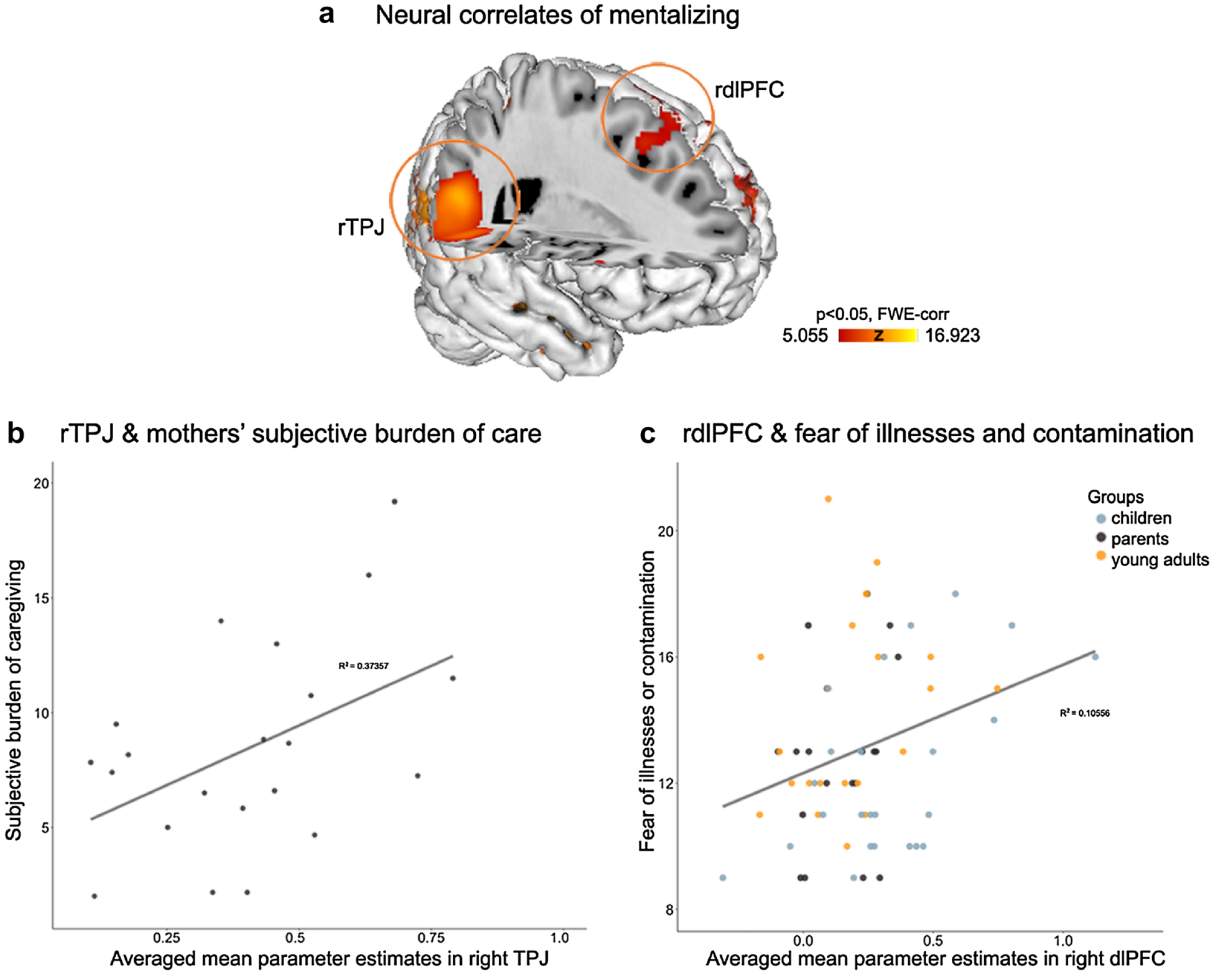
Functional brain correlates of mentalizing as assessed prior to Covid-19 onset and their associations with subjective burden and fear of illnesses and contamination reported during the early months of Covid-19. (**a**) Brain rendering for the neural correlates of mentalizing (Theory of Mind > control) across all participants (corrected for age and sex and whole brain FWE-corrected; regions of interest in right TPJ and dlPFC are circled in red). (**b**) Association between mean parameter scores during mentalizing in right TPJ and subjective burden of caregiving in mothers and (**c**) association of mean parameter scores in right dlPFC and fear of illnesses and contamination across all participants.

Across all participants, the neural correlates of mentalizing corresponded to brain regions previously associated with Theory of Mind^24^, including bilateral temporoparietal and prefrontal regions or precuneus (see peak activation reports and figure in Supplementary Table S3, Supplementary Figure S2). The multiple regression analysis revealed that activation assessed prior to Covid-19 during mentalizing in right dorsolateral prefrontal cortex was a predictor of later development of fear about illness or contamination (ß = 0.334, *t*(60) = 2.661, *p* = 0.010) constituting a significant model where dlPFC activation explained 13.9% of the variance in later reports of fear about illness or contamination (R^2^ = 0.139; adjusted R^2^ = 0.096; *F*(3,60) = 3.221, *p* = 0.029; including the covariates age and sex). Right temporoparietal junction did not enter the model as a significant predictor. When assessing the relationship between mentalizing-related activation and subjective burden, the right temporoparietal junction emerged as a significant predictor of burden (ß = 0.623, *t*(18) = 3.276, *p* = 0.004), while the dorsolateral cortex did not enter into the model. The complete model explained 41.9% of the variation in subjective burden (R^2^ = 0.419; adjusted R^2^ = 0.355; *F*(2,18) = 6.493, *p* = 0.008; including age as a covariate; Fig. 5).

## Discussion

We describe data on a small, but extensively characterized group of children and adults (N = 69, 41**♀**, age range = 7–51 years, including 26 children and their mothers), with reports across eight waves of testing, including seven assessment timepoints during the early months after Covid-19 onset in Switzerland and one assessment prior to the pandemic onset. Our findings report on mental well-being and psychosocial functioning in children and adults. The prevalence of clinically significant anxiety was 34.88%, and a 32.56% prevalence of subthreshold depression symptoms was observed across the 11 weeks. Caregiver burden was in the moderate ranges. Overall, scores of depression, anxiety and caregiver burden decreased over the course of the 11 weeks investigated. In children pre-pandemic levels of emotional and behavioral problems did not differ significantly from the average of the 11-week period during restrictions. Scores of conduct problem, hyperactivity, peer problems and overall emotional and behavioral problems in children decreased across time after Covid-19 onset. Well-being in mothers predicted mood and emotional and behavioral problems in children. In children meeting friends was a significant predictor of mood during restrictions. Additionally, neural correlates of mentalizing in prefrontal, but not temporoparietal regions, preceded the development of fear about contamination and illness across all participants. In mothers, higher neural activation in temporoparietal, but not frontal, regions during mentalizing preceded higher reports of subjective burden of care during restrictions. This may indicate that higher tendency to mentalize, usually considered beneficial for social interactions^49^ and favorable when present in mother–child dyads^50^, can be negatively associated with socioemotional functioning during prolonged stress.

Child behavior as measured by the SDQ or CBCL showed no difference when comparing pre-pandemic scores to those during restrictions, which is in line with longitudinal reports^7^ observing a relatively stable level of problem behaviors after Covid-19 onset. Based on parental reports conduct problems, hyperactivity, peer problems and the overall level of emotional and behavioral problems decreased across time in the child group studied here. Emotional problems and prosocial behavior showed no significant changes during the 11-weeks assessment period. Additionally, children’s time spent outside, and mood varied significantly. Variations in mood scores may be explained by several public holidays (Easter) around mid-restrictions. It may be possible that time spent outside during vacation allowed the meeting of friends, which was a relevant variable for increases in mood in children. Prior evidence highlights that prolonged school closure or restrictive measures are detrimental to children’s physical and mental health and can have long-lasting consequences^14^. Conversely, the present study did not identify significant changes for emotional and behavioral problems of the children comparing pre- and post-pandemic onset levels. Our findings further indicate that meeting friends predicted better mood, which is in line with prior evidence emphasizing the importance of friendships and peer relationships in developmental groups^11,45^.

Quantitative measures obtained were further supported by qualitative reports, which provide a unique insight into children’s values and further highlight sources of resilience. More specifically, children mentioned more time for leisure, sleep, family, and friends or less stress or exams as positive attributes of school closure. Negative mentions centered around restrictions affecting social contacts, prohibiting hobbies or sports, or increased stress and conflict. Interestingly, across two time points, positive mentions about returning to schools across all children solely focused on social domains (e.g., meeting friends, class, teachers again or in-person schooling), whereas negative mentions included less sleep, less free time or increased stress and homework, or restrictions. Themes reported were in line with findings of qualitative reports during Covid-19^11,45^.

Anxiety, depression and caregiver burden was high amongst adults with scores decreasing across the 11-week assessment period. Clinically significant levels of anxiety were reached at the beginning and after 7 weeks of restrictions. Furthermore, the 11-week prevalence of anxiety was 34.88%. An increase in anxiety due to Covid-19 and related restrictions has been reported previously^2,5,8,51^, however, missing pre-pandemic scores hindered a direct investigation in the present group. Mixed-effects models reflected a decrease of anxiety scores across the first months after Covid-19 onset, which is in line with similar longitudinal studies indicating a decrease following a significant early impact in affect^2,51^. Similarly, a decrease in depressive symptomatology was observed. While group average scores of depression were in the normal range, it is notable that 32.56% of all adults reported heightened depressive symptoms and 4.65% qualified for a major depressive episode at least once. These observations mirror reports of heightened depression scores in the general population during Covid-19 (e.g., retrospective reports^2^ or longitudinal data^6^). Mothers reported elevated levels of subjective burden of care (in the moderate range), which is in line with similar studies investigating parental burden during Covid-19^52^. Notably, a moderate burden of care has been associated with elevated risk for physical, psychosomatic, or mental health problems^39,52^, indicating the need for parental programs mitigating possible stress-related health consequences. The experienced subjective burden of care decreased across the early months of investigation. Distress and general health, however, did not significantly change. Longitudinal studies to date have either reported a decrease or stagnation of depression or anxiety levels for the early months following Covid-19 onset across different countries^2,29,53^. Loosen et al.^29^ for example suggest that such decreases in stress-related symptoms can partly be explained by adaptation, a phenomenon well-described in stress research^54^. Overall, first meta-analyses of studies compiling pre-/post mental health data report significant, but only small effects on anxiety and depression in adults^55^. Participant reports reflected significant changes in news consumption, reporting a higher amount of news consumed at the beginning and lower scores towards the end of the assessment period. Sex, news consumption, time spent outside or parenthood were not associated with variations in scores of anxiety, depression or distress in adults. This is somewhat surprising given prior evidence of the impact of each of these variables on mental well-being during Covid-19 (gender and parenting^44^; news consumption^2^; time spent outside^45^).

In the present study, mother–child variables were positively associated. Subjective burden of caregiving in mothers predicted emotional and behavioral problems in children, while anxiety and depression did not. This indicates that higher burden in mothers was linked to more problem behaviors in children. It is important to mention though that emotional and behavioral problems in the child were reported by the mother, thus reporting bias can’t be excluded. We further investigated the effect of the mothers’ well-being on children’s self-reported mood, demonstrating that elevated depression in mothers was associated with children’s mood ratings. Dyadic relationships are a primary vehicle for children’s learning^9^. While commonly a driver of positive effects, it may also lead to negative consequences, as demonstrated in the example of vicarious conditioned fear learning in parent–child dyads^56^. We thus hypothesize, that negative mental health in adults may negatively impact children’s well-being, possibly through learnt maladaptive coping or contagion. Increased parental stress and anxiety may lead to parental burnout^13^ or increased aggression^15^. Intergenerational care during early years lays the foundation for healthy social skill development^57^ and systemic mental health intervention programs commonly draw from this relationship^58^. Our data point towards a support of programs investing in increased parental support, which are expected to influence children’s well-being positively.

The neural correlates of mentalizing as measured prior to the pandemic in prefrontal, but not temporoparietal brain regions, preceded the development of fear about contamination and illness in all participants. In mothers, higher neural activation during mentalizing in temporoparietal, but not frontal regions was associated with higher burden of caregiving during restrictions. Activation increases in the right temporoparietal junction are commonly reported as a response to tasks of mentalizing, as this area selectively responds to observed social interactions^59^ and is part of the so-called paternal caregiver brain network^60^. Prefrontal areas are similarly engaged during tasks of mentalizing and are crucial for cognitive control processes^10^. Our data indicate that neural activation during mentalization in prefrontal cortex prior to Covid-19 may precede the development of fear of contamination and illnesses in both children and adults. The assessment of fear about contamination and illness required participants to make statements relating to the likelihood of oneself, a parent, a pet, or someone else in the world becoming sick and/or dying because of a virus or illness. Activation increases in prefrontal cortex have been linked to psychological state attributions, independent of whether they affect oneself, a relative, imagined people or animals^61^ or cognitive control (i.e., emotion regulation). A higher tendency to think about other people’s well-being, as reflected by higher mentalization-related activation in the prefrontal cortex, may thus be linked to the likelihood of developing fear about contamination and illness affecting ourselves and others.

Overall, better mentalizing has been associated with higher social competence, psychological and physiological functioning^22^, while impairments have been associated with stress and depression^23^. Increased mentalizing skills in caregivers are beneficial for child development. For example, parental mentalization has been positively associated with regulatory skills in children^62,63^, which may be protective during stressful life events^43^. However, the opposite effect may occur during stressful situations^17^. Higher levels of empathy in parents have for example been linked to better psychological and physiological health of their children, but also higher levels of inflammatory markers in the parents^63^. Moreover, higher levels of mentalizing abilities were shown to be associated with higher cortisol and heart rate reactivity in stressful situations^64^. This may temporarily be beneficial but may have a long-term negative impact depending on the intensity and duration of negative events. Our data indicate that mentalization can be negatively associated with increased burden and fear development in prolonged stressful situations.

In the present example, extensive phenotyping within individuals allow a comprehensive view and an opportunity to assess effects of time within individuals. Although the presented findings mostly align with Covid-19 literature they should be considered with caution due to the relatively small group size and less comprehensive pre-pandemic health measures. Research on the existence of potential subgroups will have to be further examined using larger and more diverse populations. An indication for possible subgroups reacting differently to stressful life events as associated with pandemics include reports of children that may in fact benefit or even thrive during restrictions^7^. A more detailed understanding of subgroups of individuals that are differently affected may increase opportunities to select the best fitting individualized treatments or prevention. Assessing direct subjective experience of the severity of impact by Covid-19 and associated restrictions would have been a valuable addition. Moreover, as the pre-pandemic assessment did not include comparable measures of mental health in the adult group, it is difficult to disentangle the effect of Covid-19 and related restrictions from pre-existing mental health symptoms. It remains to be investigated how far-reaching the herein observed negative effects on well-being are. Past work has indicated that early adversities can have an impact for life, with effects potentially being most significant in younger age and depending on the intensity of the experience^65^. An increased understanding of protective and/or risk factors and mechanisms leading to the development of stress-related psychopathologies may ultimately hold the potential to facilitate more personalized prevention and treatment strategies.

## Supplementary Information

Supplementary Information.

## Data Availability

Behavioral mean scores are included in the manuscript and neuroimaging data is provided through NeuroVault (https://identifiers.org/neurovault.collection:9780). Further information or data may be obtained from the corresponding author.
