# Supplementary material for: Mental well-being during the first months of Covid-19 in adults and children: behavioral evidence and neural precursors
Source: Sci Rep. 2021 Sep 2;11:17595. doi: 10.1038/s41598-021-96852-0 (PMC8413346; doi:10.1038/s41598-021-96852-0)
Supplement: Supplementary file 1 — Supplementary Information. [file 41598_2021_96852_MOESM1_ESM.docx]

**Supplementary information for:**

**‘Mental well-being during the first months of Covid-19 in adults and children: behavioral evidence and neural precursors’**

**Supplementary Methods**

**Additional information to the behavioral assessments pre- and during confinement**

***Assessments prior to pandemic.*** All participants previously participated in a functional magnetic resonance imaging (fMRI) study investigating socioemotional development (KIDCAT study). This study included the completion of a neuroimaging session, including functional neuroimaging (i.e., Theory of Mind or mentalizing while performing the CAToon task (Borbás et al., 2020)) and a structural scan. For children verbal and nonverbal IQ was assessed through the Matrix Reasoning and Vocabulary subtests of the fourth version of the Hamburg-Wechsler-Intelligenztest für Kinder (**HAWIK-IV**). Behavioral and emotional problems were acquired through the Strength and Difficulties Questionnaire (**SDQ**) and Child Behavior Checklist (**CBCL**) (parental responses). The children’s mothers completed the same neuroimaging session. Adults (mothers and adults unrelated to the child participants) were further assessed regarding their emotion regulation skills, general background, socioeconomic status, educational and medical history. Parents further completed assessments for parenting style, parental stress and a psychological symptoms screening.

***Detailed explanation on the assessments used during Covid-19-restrictions.*** The **STAI-6** is a short form of the state scale of the original State-Trait Anxiety Inventory (STAI) developed by Spielberger (Marteau & Bekker, 1992). It’s a self-report questionnaire to assess anxiety level as state, therefore reflecting how participants feel at the moment (Spielberger et al., 1970). Participants have to answer to 6 questions of the German version (Laux, 1981) by using a 4-point response scale ranging from 1 to 4, where the options are “not at all”, “somewhat”, “moderately”, and “very much”. A total score can be calculated by summing the scores for all items and multiply the result by 20/6 to obtain a weighted score in the same range as for STAI-20. Higher scores indicate higher levels of anxiety.

The Center for Epidemiologic Studies Depression Scale questionnaire (revised version: **CESD-R**, (Lewinsohn et al., 1997)) is a self-report instrument to screen for the presence of depressive syndrome. Participants have to respond to 20 statements of the German version of the CESD-R (Schmitt, 2016; Stadelmann et al., 2010) using a 5-point response scale depending on the temporal frequency of symptoms in the last 1 to 2 weeks. Each item is scored 0 for “not at all or less than 1 day in the past week”, 1 for “1-2 days in the past week”, 2 for “3-4 days in the past week”, 3 for “5 to 7 days in the past week” and also 3 for ”nearly every day for 2 weeks”. 9 symptoms groups can be defined: dysphoria, anhedonia, appetite, sleep, thinking, guilt, fatigue, movement and suicide (Eaton et al., 2004). A total CESD-R Score can be calculated by summing the scores for all 20 items (https://cesd-r.com).

The **GHQ-12** is a self-report instrument to screen for general psychiatric morbidity (Hankins, 2008). The questionnaire enquires the severity of psychological complaints relative to the respondent’s usual situation (Schrnitz et al., 1999), in this case compared to the previous week. Participants have to answer 12 questions of the German version of the GHQ-12 using a 4-point response scale. Here we employed the C-GHQ scoring method with an item score of 0-0-1-1. A total score is obtained summing the scores of all items, with higher scores reflecting higher levels of distress (Hankins, 2008).

The **Distress questionnaire** employed in this study derives from the Kessler Psychological Distress Scale (K10; (Kessler & Mroczek, 1994a), German version (Johannes Giesinger et al., 2008)), which is a brief behavioral screening tool to assess psychological distress with 10 questions about emotional states (e.g. feeling fidgety, hopeless, nervous, depressed) and has considered to be suitable to monitor repeated assessments. For the present study, questions from the K10 were adapted while 7 possible answers options were included. This adaptation allowed participants to indicate their emotional state in relation to their usual emotional state by selecting one of the following options: 1 - much less than usual, 2 - quite less than usual, 3 - a little less than usual, 4 - as much as usual, 5 - a little more than usual, 6 - quite a bit more than usual, 7 - much more than usual. As we did not have a direct measure of stress during the baseline phase, this adjustment serves as a proxy measurement of relative stress change during the restrictions.

The Burden Scale for Family Caregivers (BSFC; (Gräßel, 2000)) is a self-report questionnaire to assess subjective burden of family caregivers. In the short version (**BSFC-s**), respondents have to answer 10 items of the German version (Graessel et al., 2014) using a 4-point Likert scale, which ranges from 0 for “strongly disagree” to 3 for “strongly agree”. The total score ranges from 0 to 30 with higher scores indicating greater subjective burden (Graessel et al., 2014).

The **SDQ** (Goodman, 1997) is a brief behavioral screening questionnaire to assess the psychological adjustment of children and adolescents between 3 to 16 years (Goodman, 2001). Parents of the participants have to answer 25 items of the German version of the SDQ (SDQ-Deu P4-17, (Goodman, 2005; Koglin et al., 2007)) using a 3-point Likert scale. Each item is scored between 0 and 2, with the following response options: “not true”, “somewhat true” or “certainly true” (Goodman, 1997). The 25 attributes are grouped in 5 dimensions, each containing 5 items. These subscales are: conduct problems, emotional symptoms, hyperactivity, peer relationships, and prosocial behavior. Scores for each subscale are calculated by summing the scores of the 5 items. Summing the score of all the subscale except the prosocial behavior subscale generates a total difficulties score (Goodman, 1997). Higher scores in each of the 4 subscales reflect greater difficulties, whereas higher scores in the prosocial behavior subscale indicate more strength (Essau et al., 2012). Emotional and peer problems subscales can be combined into an “internalizing” subscale while conduct problems and hyperactivity subscale can be combined into an “externalizing” subscale (Goodman et al., 2010). Additionally, there is a brief impact supplement. In this section, the respondent has to answer whether he/she thinks the child has a problem. If an affirmative answer is given, other questions are asked regarding chronicity, distress, social impairment and burden to others (Goodman, 2001).

**Children’s mood** (self-report); Children have to choose between 5 different smileys in order to indicate their mood in the last days:

1
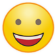
 very happy

2
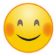
 happy

3
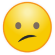
 unsure

4
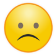
 unhappy

5
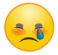
 very sad

1 is depicting a very happy, smiling face, 2 a happy, smiling face, 3 an unsure face, 4 an unhappy face and 5 a very sad face.

The **CBCL**/6-18 is a standardized questionnaire to assess children’s behavioral and emotional problems. Parents have to fill out the German version of the CBCL/6-18 by using a 3-point response scale with the following options: 0 for “not true”, 1 for “somewhat or sometimes true” and 2 for “very true or often true” (Achenbach & Ruffle, 2000). With the aid of a software package different scales can be calculated: anxious/depressed, withdrawn/depressed, somatic complaints, social problems, thought problems, attention problems, rule breaking behavior and aggressive behavior. Moreover, there are also 6 DSM-oriented scales which include affective problems, anxiety problems, somatic problems, attention deficit/hyperactivity problems, oppositional defiant problems and conduct problems (https://aseba.org).

The Fear of Illness and Virus Evaluation (**FIVE**) measures fears about contamination and illness, fears about social distancing, behaviors related to illness and virus fears and the impact of illness and virus fears (credits: Dr. Ehrenreich-May (2020), <https://adaa.org/node/5168>). Participants provided answers on a 4-point Likert scale. Children filled out the standardized Child Report Form of this questionnaire, which included identical but adjusted questions for developmental groups.

The **HAWIK-IV** (for children between 6;0 and 16;11 years) is an intelligence test based on the g-factor model to assess cognitive abilities (Daseking et al., 2007), which has also been translated and adapted for the German-speaking countries (Petermann & Petermann, 2008). It contains 15 subtests, 10 of which are needed to calculate the Full Scale IQ and the following four indices: verbal comprehension index, perceptual reasoning index, working memory index and processing speed index (Petermann & Petermann, 2008). Supplemental subtests associated with the different indices give further information (Daseking et al., 2007).

***Qualitative data assessment in children.*** included following steps: Subcategories were built based on the main topics of the answers given to the questions (1) “What do you like about spending more time at home now?” and (2) “What do you like less about spending more time at home now?” (T1-T4) and “What do you like about going back to school?” and “What do you like less or think, is a bit annoying, about going back to school?” (T5-T6). Subcategories included more time/sleep/less stress, more TV and gaming time, seeing family and friends, leisure activities (e.g., hobbies and sports), and positively perceived school related subjects (e.g., less stress, exams or school) as positive consequences (question 1), and no hobbies/sports, boredom, stress/conflicts, not being able to see family and friends, COVID-19 restrictions (e.g., not being able to meet or hug people or visit playgrounds), housework and negatively perceived school related subjects (e.g., homeschooling, homework, screen time) as negative consequences (question 2). At T5 and T6, positive consequences moreover included being able to go to school and meeting school friends again. First, the two raters coded the answers separately. Discrepancies were then discussed by the two raters until reaching a consensus. Finally, the number of answers of each subcategory was counted and displayed for overview purposes.

**Additional information to the fMRI assessments pre- and during confinement**

***fMRI CAToon task design*.**

**Supplementary Figure S1.** Timing and design for one trial of the affective (top row), cognitive (middle row) and physical causality (bottom row) conditions and the consequent decision screen including three images to choose the most likely ending from (*Figure adapted from: Borbás et al. 2020*).


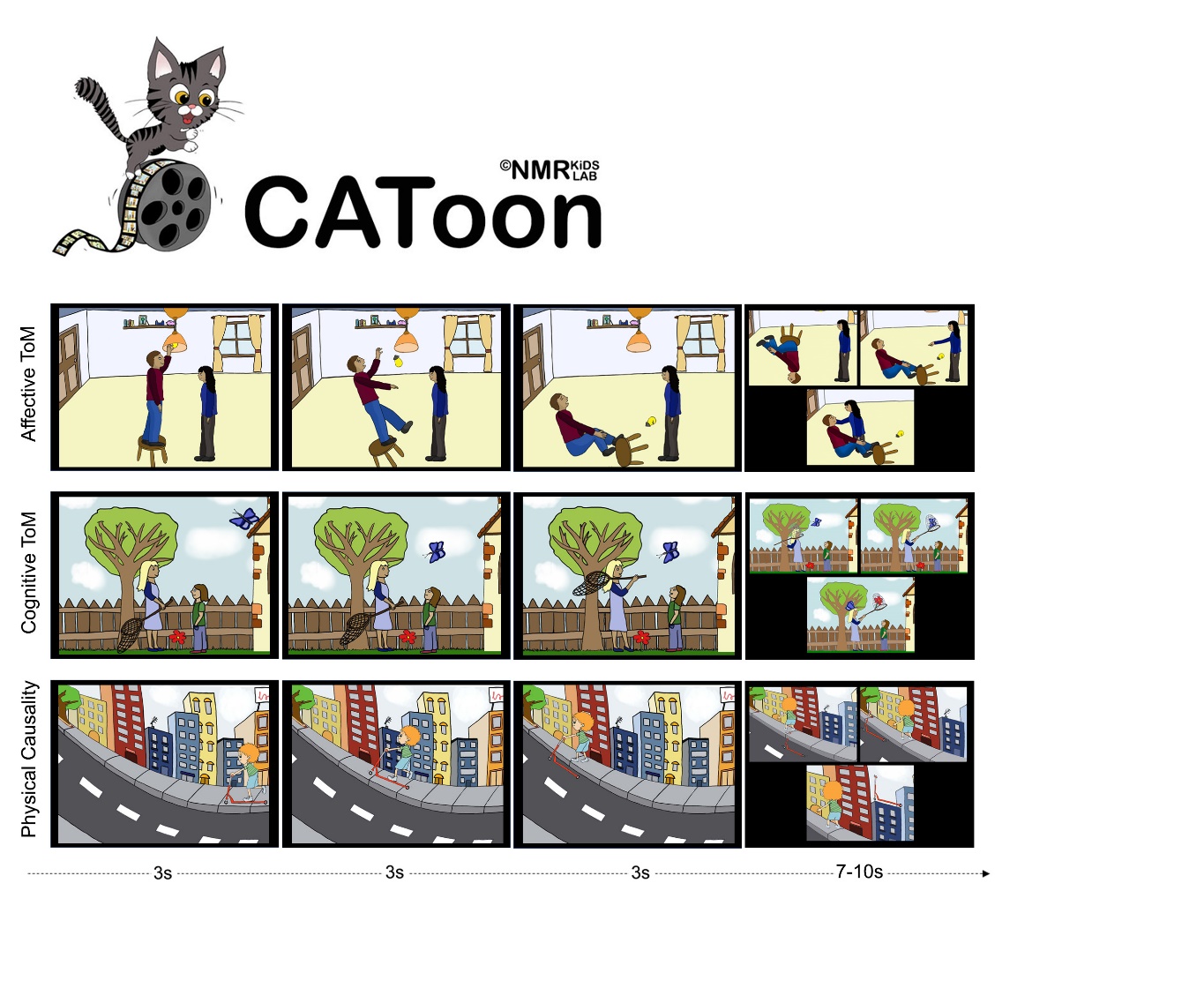
The ***C****ognitive and* ***A****ffective* ***T****heory of Mind Car****toon*** *task* **(CAToon)** is designed to measure affective and cognitive Theory of Mind (ToM) using cartoon stories. The task has an entertaining and timely design understandable and engaging for children as well as for adults. The task consists of 30 cartoon stories, representing three conditions (two ***experimental conditions*** targeting affective ToM (AT) and cognitive ToM (CT) and a ***control condition*** targeting physical causality (PC)). Each condition comprises 10 stories of similar visual complexity (i.e., backgrounds were matched across conditions). Three additional stories (one per condition) are available for practice purposes.

AT trials require participants to infer how a character would react to a fellow character’s expressed or expected emotions, whereas during CT trials participants have to assume how characters would act based on another character’s intentions or beliefs. PC trials serve as a control condition, requiring a basic understanding of cause and effect and basic physical laws.

All trials start with three images presented sequentially, followed by a single image display of three possible endings. CT trial endings consist of one correct, and two incorrect solutions. Incorrect solutions depict either a situation which would be illogical based on the storyline or physically impossible (e.g., object flies, character transforms). AT trial endings consist of two correct solutions (negative expectancy/positive expectancy) and one incorrect solution. In positive expectancy endings a character’s emotional needs are met with caring or reassuring, whereas in negative expectancy outcomes the character is scolded, ridiculed or ignored. This manipulation allows the investigation of differences in positive or negative outcome expectancy. PC trial endings have one correct and two incorrect solutions.

***MRI acquisition parameters.*** For the **fMRI task** (Theory of Mind) whole-brain T2-weighted echo-planar images were collected on a Siemens 3T Prisma MR scanner using a 20-channel head coil (transverse slice orientation, interleaved acquisition) and the following specifics: field of view=220mm, TR=2000ms, TE=30ms, 42 slices, slice thickness=2mm, voxel size=2.0x2.0x2.0mm, 333 volumes. Additionally, **structural images** were acquired and used for coregistration during image preprocessing using the following specifics: voxel size: 1.0×1.0×1.0mm; TR=1900ms; TE=3.42ms; TA=4.26; flip angle=9 degrees; field of view=256x256mm, 192 slices with a slice thickness of 1.00mm. Simultaneous multislice acquisition and dummy scans preceded image acquisition and allowed us to account for T1 equilibration effects. Dummy scans were not saved for analyses but were directly discarded.

**Supplementary Results**

**Supplementary Table S1.** Percentage of answers for each category (in %) in all groups.

| Group | Correct overall | Incorrect overall | Missing | Correct  AT | Correct  CT | Correct  PC |
| --- | --- | --- | --- | --- | --- | --- |
| Children  (n = 26) | 81.797 %  M = 24.539  SD = 2.195 | 15.000 %  M = 4.500  SD = 2.486 | 3.207 %  M = 0.962  SD = 1.536 | 93.850 %  M = 9.385  SD = 0.941 | 70.770 %  M = 7.077  SD = 1.230 | 81.150 %  M = 8.115  SD = 1.608 |
| Adults  (n = 43) | 87.830 %  M = 26.349 SD = 1.587 | 11.783 %  M = 3.535 SD = 1.609 | 0.387 %  M = 0.116 SD = 0.324 | 97.910 %  M = 9.791 SD = 0.412 | 73.490 %  M = 7.349 SD = 1.289 | 92.330 %  M = 9.233 SD = 0.812 |

Note: AT = affective ToM, CT = cognitive ToM, PC = Physical causality

**Supplementary Table S2.** One-way ANOVA for children’s emotional and behavioral problems (SDQ, CBCL) prior to and during COVID-19.

| **Measurements** |  |  | *F* | η2 |
| --- | --- | --- | --- | --- |
| **SDQ** | emotional problems | **SDQ** | 0.91 | 0.018 |
|  | conduct problems | *F*(1, 50) | 0.02 | < .001 |
|  | hyperactivity |  | 0.02 | < .001 |
|  | peer problems |  | 3.32 | 0.062 |
|  | prosocial |  | 3.16 | 0.059 |
|  | total |  | 0.03 | 0.001 |
|  |  |  |  |  |
| **CBCL** | withdrawn | **CBCL** | 0.04 | 0.001 |
|  | somatic problems | *F*(1,48) | 0.34 | 0.007 |
|  | anxious/depressed |  | 0.53 | 0.011 |
|  | social problems |  | 0.16 | 0.003 |
|  | schizoid-compulsive |  | 0.02 | <0.001 |
|  | attention problems |  | 0.01 | <0.001 |
|  | delinquent behaviour |  | 0.07 | 0.002 |
|  | aggressive behaviour |  | 1.45 | 0.029 |
|  | total |  | 1.24 | 0.025 |
| SDQ = Strengths and Difficulties Questionnaire, CBCL = Child Behavior Checklist | | | | |

| **Supplementary Table S3.** Cortical brain regions with peak activation scores for mentalizing ('*Cognitive & Affective ToM' > 'Physical Causality'*). | | | | | | | | |
| --- | --- | --- | --- | --- | --- | --- | --- | --- |
|  |  |  |  |  |  |  |  |  |
|  |  |  |  |  |  |  |  |  |
| **Brain Region** |  |  | ***P*_FWE_** | ***T*** | **k** | **MNI coordinates** | | |
| Lobe | Area | Side |  |  |  | x | y | z |
|  |  |  |  |  |  |  |  |  |
| paracentral, occipital | pre-/cuneus, cingulum, lingual | R/L | <0.001 | 16.9 | 5207 | 6 | -52 | 46 |
| temporal, occipital | temporal, calcarine, fusiform, angular, supramarginal gyrus, frontal, calcarine, lingual, supramarginal | R | <0.001 | 14.3 | 5860 | 48 | -52 | 16 |
| temporal, occipital, frontal | temporal pole, occipitotemporal, frontal, fusiform, insula | L | <0.001 | 13.4 | 5663 | -50 | -58 | 20 |
| frontal | frontal, anterior cingulum | R/L | <0.001 | 10.2 | 1207 | 6 | 54 | 16 |
| temporal | inferior temporal, lingual, fusiform, para-/hippocampus | L | <0.001 | 9.63 | 428 | -38 | -40 | -18 |
| temporal | inferior temporal, cerebelum, fusiform, parahippocampal | R | <0.001 | 8.9 | 246 | 42 | -44 | -22 |
| frontal | medial frontal, gyrus rectus | R/L | <0.001 | 7.9 | 262 | 2 | 50 | -18 |
| frontal | frontal, supplementary motor area | R | <0.001 | 7.66 | 271 | 14 | 36 | 60 |
| frontal | mid frontal, precentral | L | <0.001 | 6.84 | 169 | -42 | 12 | 56 |
| frontal | inferior frontal | R | <0.001 | 6.67 | 64 | 54 | 28 | -2 |
| frontal | insula, inferior frontal | R | 0.001 | 6.63 | 44 | 38 | 22 | -18 |
| frontal | superior frontal | L | <0.001 | 6.62 | 59 | -6 | 42 | 56 |
| frontal | inferior frontal | R | <0.001 | 5.92 | 53 | 34 | 14 | 26 |
| temporal | para-/hippocampus | L | 0.005 | 5.71 | 18 | -24 | -16 | -16 |
| temporal | hippocampus | R | 0.008 | 5.7 | 13 | 22 | -8 | -16 |
| temporal | inferior temporal | L | 0.017 | 5.67 | 6 | -40 | -14 | -28 |
| frontal | superior frontal | L | 0.005 | 5.55 | 18 | -12 | 50 | 46 |
| temporal | hippocampus | L | 0.03 | 5.28 | 2 | -30 | -6 | -22 |
|  |  |  |  |  |  |  |  |  |
|  |  | L=left |  |  | k = cluster size | | | |
|  |  | R=right |  |  |  | |  |  |
|  |  |  |  |  |  |  |  |  |
|  |  |  |  |  |  |  |  |  |


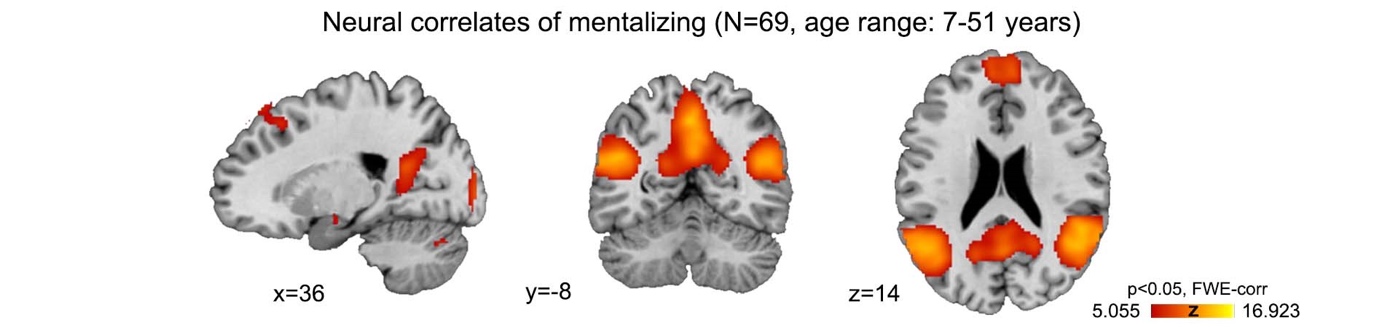


**Supplementary Figure S2.** Brain renderings for the neural correlates of mentalizing (Theory of Mind > control) across all participants (corrected for age and sex and whole brain FWE-corrected; p < 0.05)

**REFERENCES**

Abler, B., & Kessler, H. (2009). Emotion regulation questionnaire–Eine deutschsprachige Fassung des ERQ von Gross und John. *Diagnostica, 55*(3), 144-152.

Achenbach, T. M., & Ruffle, T. M. (2000). The Child Behavior Checklist and related forms for assessing behavioral/emotional problems and competencies. *Pediatrics in review, 21*(8), 265-271.

Andershed, H., Hodgins, S., & Tengström, A. (2007). Convergent validity of the youth psychopathic traits inventory (YPI) association with the psychopathy checklist: youth version (PCL: YV). *Assessment, 14*(2), 144-154.

Andershed, H. A., Kerr, M., Stattin, H., & Levander, S. (2002). Psychopathic traits in non-referred youths: A new assessment tool.

Borbás, R., Fehlbaum, L., Rudin, U., Stadler, C., & Raschle, N. (2020). Neural correlates of Theory of Mind in children and adults using CAToon-introducing an open-source child-friendly neuroimaging task.

Boulet, J., & Boss, M. W. (1991). Reliability and validity of the Brief Symptom Inventory. *Psychological Assessment: a journal of consulting and clinical psychology, 3*(3), 433.

Daseking, M., Petermann, U., & Petermann, F. (2007). Intelligenzdiagnostik mit dem HAWIK-IV. *Kindheit und Entwicklung, 16*(4), 250-259.

Davis, M. H. (1980). A multidimensional approach to individual differences in empathy.

Derogatis, L. R., & Melisaratos, N. (1983). The brief symptom inventory: an introductory report. *Psychological medicine, 13*(3), 595-605.

Eaton, W. W., Smith, C., Ybarra, M., Muntaner, C., & Tien, A. (2004). Center for Epidemiologic Studies Depression Scale: review and revision (CESD and CESD-R).

Essau, C. A., Olaya, B., Anastassiou‐Hadjicharalambous, X., Pauli, G., Gilvarry, C., Bray, D., O'callaghan, J., & Ollendick, T. H. (2012). Psychometric properties of the Strength and Difficulties Questionnaire from five European countries. *International Journal of Methods in Psychiatric Research, 21*(3), 232-245.

Essau, C. A., Sasagawa, S., & Frick, P. J. (2006). Psychometric properties of the Alabama parenting questionnaire. *Journal of Child and Family Studies, 15*(5), 595-614.

Frick, P. J. (1991). *The Alabama Parenting Questionnaire*.

Garnefski, N., Kraaij, V., & Spinhoven, P. (2001). Negative life events, cognitive emotion regulation and emotional problems. *Personality and Individual differences, 30*(8), 1311-1327.

Garnefski, N., Rieffe, C., Jellesma, F., Terwogt, M. M., & Kraaij, V. (2007, Feb). Cognitive emotion regulation strategies and emotional problems in 9 - 11-year-old children: the development of an instrument. *Eur Child Adolesc Psychiatry, 16*(1), 1-9. https://doi.org/10.1007/s00787-006-0562-3

Giesinger, J., Rumpold, G., & Schussler, G. (2008). German version of the K10-Screening Scale for psychological distress. *PSYCHOSOMATIK UND KONSILIARPSYCHIATRIE, 1*(2), 103-110.

Giesinger, J., Rumpold, M. G., & Schüßler, G. (2008). Die k10-screening-skala für unspezifischen psychischen distress. *Psychosomatik und Konsiliarpsychiatrie, 2*(2), 104-111.

Goodman, A., Lamping, D. L., & Ploubidis, G. B. (2010). When to use broader internalising and externalising subscales instead of the hypothesised five subscales on the Strengths and Difficulties Questionnaire (SDQ): data from British parents, teachers and children. *Journal of abnormal child psychology, 38*(8), 1179-1191.

Goodman, R. (1997). The Strengths and Difficulties Questionnaire: a research note. *Journal of child psychology and psychiatry, 38*(5), 581-586.

Goodman, R. (2001). Psychometric properties of the strengths and difficulties questionnaire. *Journal of the American Academy of Child & Adolescent Psychiatry, 40*(11), 1337-1345.

Goodman, R. (2005). Fragebogen zu Stärken und Schwächen (SDQ-Deu). *Abgerufen am, 1*, 2019.

Graessel, E., Berth, H., Lichte, T., & Grau, H. (2014, Feb 20). Subjective caregiver burden: validity of the 10-item short version of the Burden Scale for Family Caregivers BSFC-s. *BMC Geriatr, 14*, 23. https://doi.org/10.1186/1471-2318-14-23

Gräßel, E. (2000). Warum pflegen Angehörige? Ein Pflegemodell für die häusliche Pflege im höheren Lebensalter. *Zeitschrift für Gerontopsychologie und-psychiatrie*.

Grob, A., & Hagmann-von Arx, P. (2012). *Fallbuch IDS: die Intelligence and Development Scales in der Praxis*. Hogrefe Verlag.

Gross, J. J., & John, O. P. (2003, Aug). Individual differences in two emotion regulation processes: implications for affect, relationships, and well-being. *J Pers Soc Psychol, 85*(2), 348-362. http://www.ncbi.nlm.nih.gov/pubmed/12916575

Hankins, M. (2008). The reliability of the twelve-item general health questionnaire (GHQ-12) under realistic assumptions. *BMC public health, 8*(1), 1-7.

Judy O. Berry, W. H. J. (1995). The Parental Stress Scale: Initial Psychometric Evidence.

Kessler, R., & Mroczek, D. (1994a). Final versions of our non-specific psychological distress scale. *Ann Arbor, MI: Survey Research Center of the Institute for Social Research, University of Michigan*.

Kessler, R., & Mroczek, D. (1994b). Final versions of our non-specific psychological distress scale. *Memo dated March, 10*, 1994.

Koglin, U., Barquero, B., Mayer, H., Scheithauer, H., & Petermann, F. (2007). Deutsche Version des Strengths and Difficulties Questionnaire (SDQ-Deu). *Diagnostica, 53*(4), 175-183.

Köhler, D., Kuska, S. K., Schmeck, K., Hinrichs, G., & Fegert, J. (2010). Deutsche Version des Youth-Psychopathic-Traits-Inventory (YPI). *Klinisch-psychiatrische Ratingskalen für das Kindes-und Jugendalter*, 478-482.

Laux, L. (1981). Das State-Trait-Angstinventar (STAI): Theoretische Grundlagen und Handanweisung.

Lewinsohn, P. M., Seeley, J. R., Roberts, R. E., & Allen, N. B. (1997). Center for Epidemiologic Studies Depression Scale (CES-D) as a screening instrument for depression among community-residing older adults. *Psychology and aging, 12*(2), 277.

Louie, A. D., Cromer, L. D., & Berry, J. O. (2017). Assessing parenting stress: Review of the use and interpretation of the parental stress scale. *The Family Journal, 25*(4), 359-367.

Marteau, T. M., & Bekker, H. (1992). The development of a six-item short-form of the state scale of the Spielberger State—Trait Anxiety Inventory (STAI). *British Journal of Clinical Psychology, 31*(3), 301-306. https://doi.org/10.1111/j.2044-8260.1992.tb00997.x

Minnis, H., Rabe‐Hesketh, S., & Wolkind, S. (2002). Development of a brief, clinically relevant, scale for measuring attachment disorders. *International Journal of Methods in Psychiatric Research, 11*(2), 90-98.

Petermann, F., & Petermann, U. (2008). German WISC-IV. *Kindheit und Entwicklung, 17*(2), 71-75.

Schmitt, A. (2016). *German version of the Center for Epidemiologic Studies Depression Scale Revised (CESD-R)*. https://doi.org/10.13140/RG.2.1.4950.5365

Schrnitz, N., Kruse, J., & Tress, W. (1999). Psychometric properties of the General Health Questionnaire (GHQ‐12) in a German primary care sample. *Acta Psychiatrica Scandinavica, 100*(6), 462-468.

Schröder, M., Fux, E., Lüdtke, J., Izat, Y., Bolten, M., & Schmid, M. (2019). German Version of the Relationship Problems Questionnaire: Effective Screening for Attachment Disorder. *Psychopathology, 52*(6), 334-345.

Stadelmann, S., Perren, S., Kölch, M., Groeben, M., & Schmid, M. (2010). Psychisch kranke und unbelastete Eltern. *Kindheit und Entwicklung*.
